# Effectiveness of a Virtual Reality rehabilitation in stroke patients with sensory-motor and proprioception upper limb deficit: A study protocol

**DOI:** 10.1371/journal.pone.0307408

**Published:** 2024-08-12

**Authors:** Sara Ventura, Alessia Tessari, Sara Castaldini, Elisabetta Magni, Andrea Turolla, Rosa Baños, Giada Lullini

**Affiliations:** 1 Instituto Polibienestar, University of Valencia, Valencia, Spain; 2 Department of Psychology, University of Bologna, Bologna, Italy; 3 UOC di Medicina Riabilitativa e Neuro-riabilitazione, IRCCS Istituto delle Scienze Neurologiche di Bologna, Azienda USL Bologna, Bologna, Italy; 4 Department of Biomedical and Neuromotor Sciences–DIBINEM, University of Bologna, Bologna, Italy; Kio University, JAPAN

## Abstract

**Introduction:**

Stroke is the second leading cause of death in Europe. In the case of stroke survival (almost 70%), only 25% of patients recover completely, while the remaining 75% will undergo a rehabilitation phase that varying from months to years. The primary outcomes of a stroke involve motor impairment in the upper limbs, resulting in a partial or complete inability to move the limb on the right or left side, depending on the affected hemisphere. Furthermore, the motor deficit distorts the proprioception of the body and the embodiment ability of the injured limb. This could be rehabilitated through the paradigm of body illusion that modulates the motor rehabilitation. The present protocol aims to investigate the effectiveness of a Virtual Reality system for sensorimotor and proprioception upper limb deficit compared to a traditional upper limb rehabilitation program.

**Method:**

This study has a randomized and controlled design with control and experimental groups, and 4 measurement times: pre-intervention, immediately after the intervention, and two follow-ups (at 6 and 12 months). The inclusion criteria are: (a) Being 18 to 85 years old, both males and females; (b) Suffering from ischemic or haemorrhagic stroke; (c) The stroke event must have occurred from two to eighteen months before recruitment; (d) Patients must have moderate to severe upper limb motor deficit, and the alteration of sensorimotor and proprioception abilities of the injury upper limb; (e) Patients must understand and sign the written consent for enrolment. The rehabilitation last four weeks with three sessions per week at Bellaria Hospital of Bologna (Italy). The VR protocol uses two types of technology: immersive and non-immersive, and the control group follow the traditional rehabilitation program.

## 1. Introduction

According to the European Cardiovascular Disease Statistics, stroke is the second leading cause of death in Europe. It mainly occurs after age 55, with 75% of cases in people over 65 [1]. After a stroke, 20–30% of people die in the first month from the event and 40–50% within the first year. For those who survive, only 25% experience full recovery, with the remaining 75% entering a rehabilitation phase that spans from several weeks to several months or even years. Published randomized control trials that adopted various rehabilitative programs including technology have demonstrated that, at the end of the rehabilitation period, the patient may experience full recovery or remain in a state of permanent disability [2–4].

The primary outcomes of a stroke encompass motor impairment in the upper limbs, leading to a partial or total inability to move either the right or left limb based on the affected hemisphere [5]. In particular, patients experience difficulty performing reaching tasks due to a lack of motor coordination or an inability to control grip and finger strength when manipulating objects [6]. In everyday life, the impairment in the upper limbs manifests as an inability to independently eat, dress, maintain personal hygiene, and engage in other self-care activities, resulting in a reliance on a caregiver. Consequently, consistent rehabilitation to restore motor skills becomes crucial for both the patient and their family members [7].

From a neuroscience standpoint, deficiencies in the planning, preparation, and execution of movements may arise due to impairment in the primary motor cortices or areas responsible for praxis control, particularly those situated in the parietal regions [8–11], which alter proprioceptive and kinaesthetic signals and the perception of peripersonal space [12]. Proprioception refers to the sense of the relative position of one’s own body parts and strength of effort being employed in movement, which can be impaired after a stroke [13]. As a result, patients present distorted body representations and an alteration in the sense of embodiment, in terms of ownership, location, and agency. In particular, people who have survived a stroke experience apparent changes in their bodies, such as altered sensations, impaired limb function, uncoordinated movements and disrupted proprioception [14–16]. Furthermore, sensation, emotions, and perception are some of the characteristics of self-consciousness assimilated into the physical body thanks to the sense of embodiment [17], and that in stroke patients is compromised. The somatosensory deficit that affects this sample of patients refers to an impairment or loss of sensation related to touch, pressure, temperature, or pain on the side of the body affected by the stroke. This deficit occurs due to damage to the brain regions responsible for processing sensory information and patients maintain various delusional beliefs regarding the ownership of their paralyzed limbs [14, 18, 19]. This lack of awareness or recognition of the body can impact the sense of ownership and may lead to difficulties in coordinating movements or adjusting to changes in body perception [20].

Taking into account the concept of neural plasticity, which refers to the brain’s capacity to adapt and facilitate functional activities [21], recent research indicates that intervening with the illusion of the bodily self in hemiplegic patients could enhance the rehabilitation of the affected limb [22, 23]. One of the methods to induce the limb illusion is the mirror box. In this apparatus, the patient sees the reflection of their healthy arm in the mirror, and if the illusion is successfully induced, they perceive the reflected arm as their own instead of the injured one [24]. This process could be possible because the embodiment of the hand reflected by the mirror would improve the reorganization of body representation in patients with post-stroke motor deficits (Tosi et al., 2018). The paradigm of the body illusion was then translated into the Virtual Reality (VR) system [25].

Over the past decades, VR has been widely used in upper limb motor rehabilitation following a stroke, yielding significant results compared to a traditional rehabilitation program [26, 27]. VR is an advanced technology that provides interactive environments that reproduce the surrounding reality. It is divided into immersive and non-immersive systems; the former projects the three-dimensional environment through a Head Mounted Display wear by participants, often incorporate other sensory feedback, such as spatial audio and sometimes haptic feedback, to enhance the feeling of presence within the virtual environment [28, 29]; the latter, on the other hand, projects the virtual environment into a screen and the patient can interact with it through devices such as joysticks or cyber-gloves [30]. Previous literature in the field of post-stroke rehabilitation through VR demonstrated its effectiveness in improving neuroplasticity and motor recovery thanks to several aspects such as real-time feedback, customization of exercises according to the patient’s cognitive and motor abilities, the immersive and interactive experience that the technology offers, and a faithful simulation of real-world activities [31–33]. VR has demonstrated its effectiveness in upper limb rehabilitation, but little is known about how integrating the illusion of one’s limb can benefit rehabilitation. Previous study found that inducing a strong feeling of ownership of a virtual body that could perform movements of any complexity and duration might contribute to restoring motor functions in stroke patients. In this line, a recent systematic review demonstrated the significant modulating role of body ownership illusion through VR to restore motor abilities after stroke [34]. Furthermore, the VR technique can provide interaction between virtual objects and body motion using motion tracking. This technique has proven to be suitable for proprioception rehabilitation due to its ability to manipulate the visual feedback of virtual objects [35]. The VR proprioceptive paradigm is innovative because this type of exercise system cannot be easily provided in traditional therapy. That is, for healthy individuals, the central nervous system integrates multiple modes of sensory information, especially vision and proprioception, to perform motor tasks. In stroke patients, however, the integration of multiple sensory inputs is impaired, and they can only rely on intact visual information rather than somatosensory input [36]. In this case, visual influence becomes predominant when afferent input from other sources is reduced, and the predominant influence of visual input constitutes a natural compensatory strategy for coping with initial stroke damage [37]. According to the theory of neural plasticity cited above, the brain has the ability to reorganize itself by forming new neural connections in response to sensory inputs and repetitive practice [21]. Even if the sensory feedback in VR is an illusion, it can still promote neural plasticity similarly to real sensory signals [38]. For this reason, VR could be an efficacious medium to improve the proprioception abilities and the motor rehabilitation after stroke. However, new study protocols are needed.

Therefore, considering the high incidence of stroke, the disability it entails, and the need for timely and constant rehabilitation, together with the promising data of VR as an effective tool for sensorimotor and proprioceptive rehabilitation, the present project aims to investigate the effectiveness of a VR system for sensorimotor and proprioception upper limb deficit compared to a traditional upper limb rehabilitation program.

## 2. Objectives and hypothesis

Considering the sensorimotor deficits after a stroke and the relate difficulty to build an internal representation of the own body, plus the significative feature of VR to induce a body illusion, the present protocol aims to investigate the effectiveness of a Virtual Reality rehabilitation program to restore the sensorimotor and proprioceptive abilities of the injured upper limb after a stroke, compared with the treatment as usual (TAU). Moreover, the feature of the exergame of VR would engage the patients and motivate them to pursue the rehabilitation program and overcome the drop-out from the rehabilitation.

### 2.1. Hypothesis 1

VR rehabilitation programs will generally be as effective as TAU in increasing the motor abilities of the injured upper limb after a stroke.

### 2.2. Hypothesis 2

The VR rehabilitation program will be more effective than TAU in the proprioception abilities of the injured upper limb.

### 2.3. Hypothesis 3

The TAU will be the least satisfying rehabilitation program for the patient, with a higher probability of abandonment compared to VR.

### 2.4. Hypothesis 4

The same results will be found across the follow-up assessments (6 and 12 months after intervention).

## 3. Method

The Local Ethics Committees approved the project protocol (ASL_BO n. 0115481 provided on 18/10/2022) before commencing the recruitment and registered to ClinicalTrials.com (ID: NCT06164054) The study is performed according to the principle of the Helsinki Declaration. The study results will be disseminated in peer-reviewed scientific journals and in abstract format at scientific events.

### 3.1. Study design

**Fig 1:**
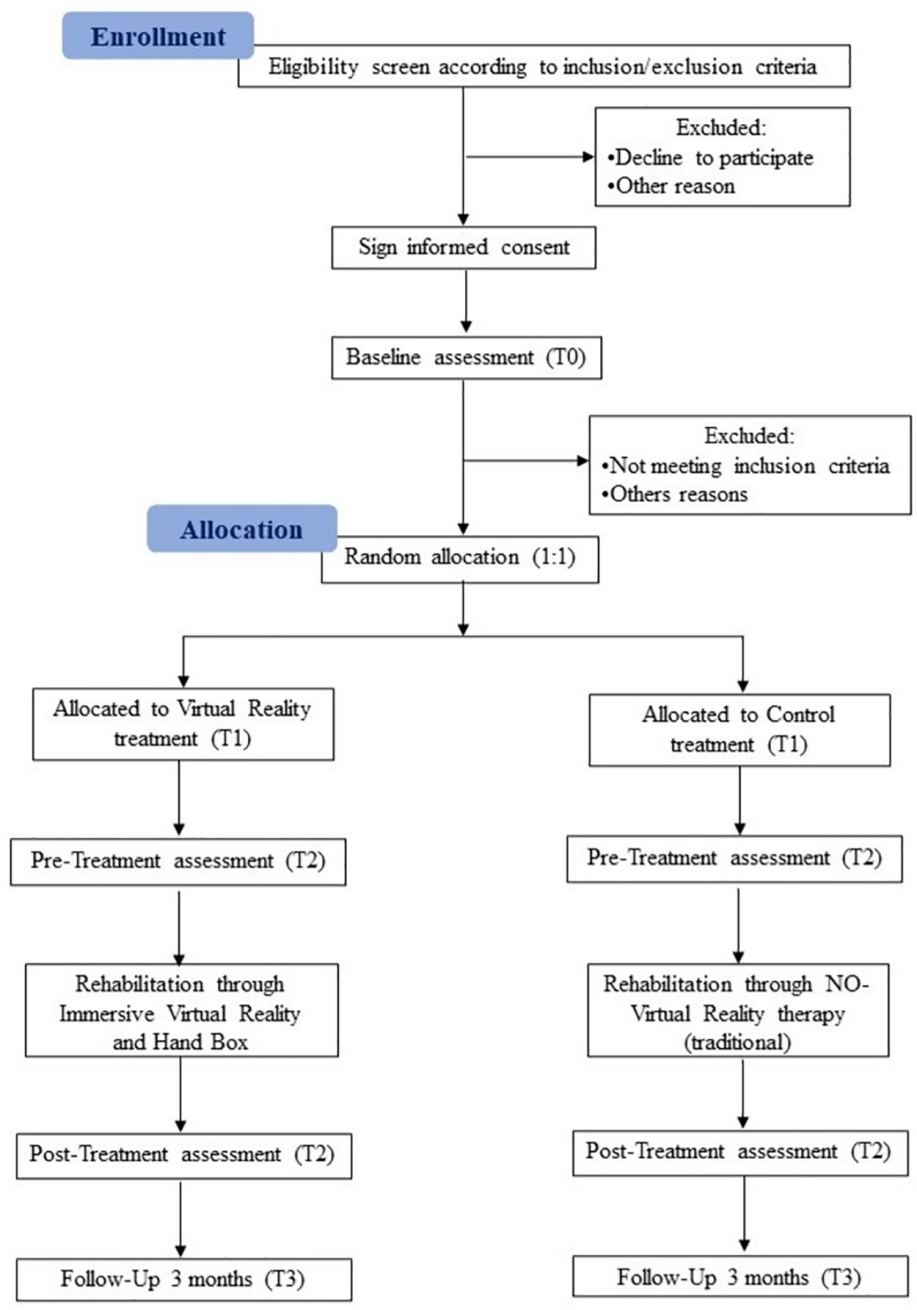
CONSORT flowchart.

A single-blind (the patient is blind to group assignment) two-arm randomized controlled trial is proposed with a blocking randomization [39]. Participants who have had a stroke are randomly allocated to: (1) 4 weeks traditional rehabilitation program (control group), or (2) 4 weeks to Virtual Reality rehabilitation program (experimental group). The study design presents 4 measurement times: pre-intervention, immediately after the intervention, and two follow-ups (at 6 and 12 months; see Fig 1). The rehabilitation programs, both experimental and control, will be performed at the Bellaria Hospital of Bologna (Italy). Moreover, participants must sign written consent forms for study participation and personal data handling and management.

### 3.2. Participants

The participants’ groups involve people with stroke selected according to inclusion and exclusion criteria and keen to participate in the study. Patients are enrolled in the Neurorehabilitation Unit of the Institute for Neurological Sciences of Bologna (Istituto delle Scienze Neurologiche di Bologna, ISBN). Furthermore, the potential participants are informed about participation in the study during the check-up visits scheduled by ISNB medical doctors referring the patients.

#### 3.2.1. Inclusion criteria

Patients must have all the following inclusion criteria: (a) Being 18 to 85 years old, both males and females; (b) Suffering from ischemic or haemorrhagic stroke; (c) The stroke event must have occurred from two to eighteen months before recruitment [40]; (d) Patients must have moderate to severe upper limb motor deficit established by a score of ≤ 80 on the Motricity Index [41], and the alteration of sensorimotor and proprioception abilities of the injury upper limb, established by the failure in 3 proofs up to 4 of the Thumb Location Test [19]; (e) Patients must understand and sign the written consent for enrolment.

#### 3.2.2. Exclusion criteria

Patients presenting at least one of the following exclusion criteria are not eligible to be enrolled in the study: (a) Severe psychiatric (e.g., psychosis, depression, apathy) and behavioral disorders (e.g., severe psychomotor agitation), cognitive disorders, or a state of confusion defined by temporal and/or spatial disorientation detected during an ordinary conversation. A simple confusion state assessment test (4AT) is administered in case of doubt [42]; (b) Severe upper limb motor deficit with the following score at Motricity Index Scale: gripper <11, elbow flexion <14, shoulder abduction <14 [41]; (c) Verbal comprehension ability with a score of <2 at Token Test [43]; (d) Severe spatial neglect with a score of >3 at Barrage test [44].

### 3.3. Assessment

The basic information, including age, sex, lesion side of the brain, stroke type, and duration after stroke onset, was recorded. The clinical outcomes include motor assessment, which examines the motor function of the patients’ injured upper limb, as well as neuropsychological assessment to evaluate cognitive abilities for the eligibility criteria and to investigate if the rehabilitation program could also impact the patient’s cognition. Additionally, patient’s self-efficacy and satisfaction toward the treatment are also evaluate.

#### 3.3.1. Motor assessment

- The *Fugl-Meyer* is subscale included 33-item upper limb activities. Each item was rated on a 0 to 2 ordinal scale. The maximum score of the FMA-UL subscale was 66 [45].
- The *Motricity Index* for upper limb with a scores ranging from 0 to 100. It evaluated the shoulder abduction, the elbow flexion, and the “grip and pinch” abilities [41].
- The *Box and Blocks test* which contains 150 wooden cube blocks (1 inch). The participants were told to move one-by-one blocks as many as possible from a rectangular box container to the other of equal size within 60 seconds. Both hands’ scores of the BBT were calculated, respectively, by the number of blocks transferred [46].

#### 3.3.2. Neuropsychological assessment

- Proprioception regarding the perception of patient’s body:
  - Multidimensional Assessment of Interoceptive Awareness (MAIA) [47]: the self-report assesses the following eight interoceptive dimensions: (1) noticing (i.e., tendency to be aware of one’s body sensations, regardless of their (dis)comfort); (2) not-distracting (i.e., tendency to not ignore uncomfortable sensations in the body or pain), (3) not-worrying (i.e., tendency to not worry about uncomfortable sensations in the body or pain); (4) attention regulation (i.e., ability to pay attention to sensations from the body); (5) emotional awareness (i.e., extent to which emotions are perceived as connected to bodily sensations); (6) self-regulation (i.e., ability to use attention to sensations from the body to regulate distress); (7) body listening (i.e., listening actively to the body for insight); and (8) trusting (i.e., degree to which the body is experienced as safe).
  - Thumb Location Test [19] to evaluate the ability of individuals to accurately locate their thumbs without visual cues. During the test, the individual typically closes their eyes or is blindfolded, and the examiner moves the person’s thumb to different positions. The individual is then asked to indicate the location of their thumb by pointing to it with their other hand or verbally describing its position and it is scored from 0 = not accurate to 2 = completely accurate.
  - Rubber Hand Illusion [48] it is a self-report questionnaire that evaluate the participants’ ability to perceive a rubber hand as his/her own in term of ownership, location and agency. The test is administered after the rubber hand induction by a professional with patient’s injury limb. The scale is score from -3 (not at all) to +3 (completely).
- The Short screening test for ideo-motor apraxia (STIMA) [49] based on the presentation of separated lists of intransitives 18 meaningfulness gestures and 18 meaninglessness gesture score from 1 when the participant successfully imitates on the first attempt, and 2 at the second.
- The Raven progressive matrices [50] to measure the patient’s abstract reasoning and non-verbal intelligence. It consists of a series of visual pattern problems, where participants are asked to identify the missing piece that completes a pattern.
- The Trials Making Test (A and B) [51] to evaluate patient’s attention. It is composed of part 1 with a sheet of paper containing circles numbered from 1 to 25. The task is to connect the circles in numerical order as quickly as possible, and part 2 more complex and involves connecting circles that alternate between numbers and letters (e.g., 1-A-2-B-3-C, and so on) in ascending order. The individual is instructed to switch between numbers and letters while connecting the circles.
- Memory ability:
  - Corsi Test (visuospatial) [52] used to measure visuospatial short-term memory and spatial span. During the assessment, the participant is presented with a series of blocks, typically arranged in a random pattern and often mounted on a board. The examiner taps a sequence of blocks, and the participant is then asked to reproduce the sequence in the same order. The test progresses in difficulty by increasing the length of the sequences. Performance is typically measured by the longest sequence of blocks that the participant can accurately reproduce.
  - Monaco Test (or digit span forward and backward) [53] to assess the participant’s short-term memory and working memory capacity. It involves the participant repeating sequences of numbers, either forwards or backwards, immediately after they are presented. In the "Digit Span Forward" task, the participant is given a sequence of numbers and is asked to repeat them in the same order. For example, if the examiner says "2, 5, 7," the participant would respond with "2, 5, 7”. In the "Digit Span Backward" task, the participant is given a sequence of numbers and is asked to repeat them in the reverse order. Using the same example, if the examiner says "2, 5, 7," the participant would respond with "7, 5, 2." The test ends when the participant repeats in a wrong way for two consecutive times.
- Token Test [43] to evaluate the participant’s language comprehension. During the assessment, the participant is presented with a series of commands that involve manipulating tokens (e.g., coins, chips) according to specific instructions. The complexity of the commands gradually increases throughout the test. Performance on the Token Test is evaluated based on the participant’s ability to accurately follow the instructions, manipulate the tokens according to the commands, and demonstrate comprehension of various linguistic concepts such as spatial relationships, object attributes, and logical sequencing.
- Visuo-spatial ability:
  - Barrage [44] peripersonal neglect and measure the patient’s spatial and selective attention abilities.
  - Visual object and space perception (VOSP) [54] assessment tool used to evaluate various aspects of visual perception, including object recognition and spatial processing. The test includes “object perception task” that assess the ability to recognize and discriminate between different objects. Examples include matching identical objects, discriminating between similar objects, and identifying fragmented objects; and “space perception task” to evaluate the spatial processing abilities, such as judging the orientation of objects in space, detecting spatial relationships between objects, and perceiving spatial patterns.
- Self-efficacy state:
  - Functional Independence Measure (FIM) [55] to evaluate the patients’ self-efficacy after a stroke. The test involves six aspects of daily function: self-care, sphincter control, transfer, locomotion, communication, and social cognition ability. It was made of 18 items, and each item was graded on a 1 to 7 ordinal scale. The total score ranged from 7 to 126.
  - Stroke Self-Efficacy (SSEQ) [56] is a self-report measure designed to assess stroke survivors perceived self-efficacy in managing the various challenges they face during stroke recovery. Self-efficacy refers to an individual’s belief in their ability to successfully perform specific tasks and achieve desired outcomes in a given situation. It is composed by 13 items with a score between 0 = not at all to 3 = completely.
- Client Satisfaction Questionnaire (CSQ-8) [57], it is a self-report questionnaire that measure the patients’ satisfaction toward the rehabilitation program. It is composed by 8 items ranged from -1 = not at all to +4 = completely.

Table 1 shows the protocol’s assessment.

**Table 1:** Protocol’s assessment.

Patients’ abilities	Test	Screening (T0)	Pre-test (T1)	Post-test (T2)	Follow-Up (T3)
*Motor*	Fugl Meyer (Platz et al., 2005)		x	x	x
	Motricity Index (Bohannon, 1999)	x			
	Box and Blocks (Mathiowetz et al., 1985)		x	x	x
*Proprioception*	Multidimensional Assessment of Interoceptive Awareness (MAIA) (Mehling et al., 2015)		x	x	x
	Thumb Location Test (Rand, 2018)	x			
	Rubber Hand Illusion (Romano et al., 2021)		x	x	x
*Apraxia*	Short screening test for ideo-motor apraxia (STIMA) (Tessari et al., 2015)		x		
*Intelligence*	Raven progressive matrices (Carpenter et al., 1990)		x		
*Attention*	Trials Making Test (A and B) (Tombaugh, 2004)		x		
*Memory*	Corsi Test (visuospatial) (Piccardi et al., 2013)		x		
	Monaco Test (span forward and backward) (Monaco et al., 2013)		x		
*Language comprehension*	Token Test (De Renzi et al., 1962)	x			
*Visuo-spatial*	Barrage (Albert, 1973)	x			
	Visual object and space perception (VOSP) (Quental et al., 2013)		x		
*Self-efficacy*	Functional Independence Measure (FIM) (Linacre et al., 1994)		x	x	x
	Stroke Self-Efficacy (SSEQ) (Dallolio et al., 2016)		x	x	x
*Satisfaction*	Client Satisfaction Questionnaire (CSQ-8) (Attkisson and Zwick, 2006).			x	

### 3.4. Interventions

The rehabilitation will take place in the rehabilitative room at Bellaria Hospital of Bologna (Italy), which is intended for physical and neurological rehabilitation for people with brain injuries. It is equipped by traditional therapeutic tools and the Virtual Reality apparatus.

#### 3.4.1. Virtual Reality rehabilitation setting

**Fig 2:**
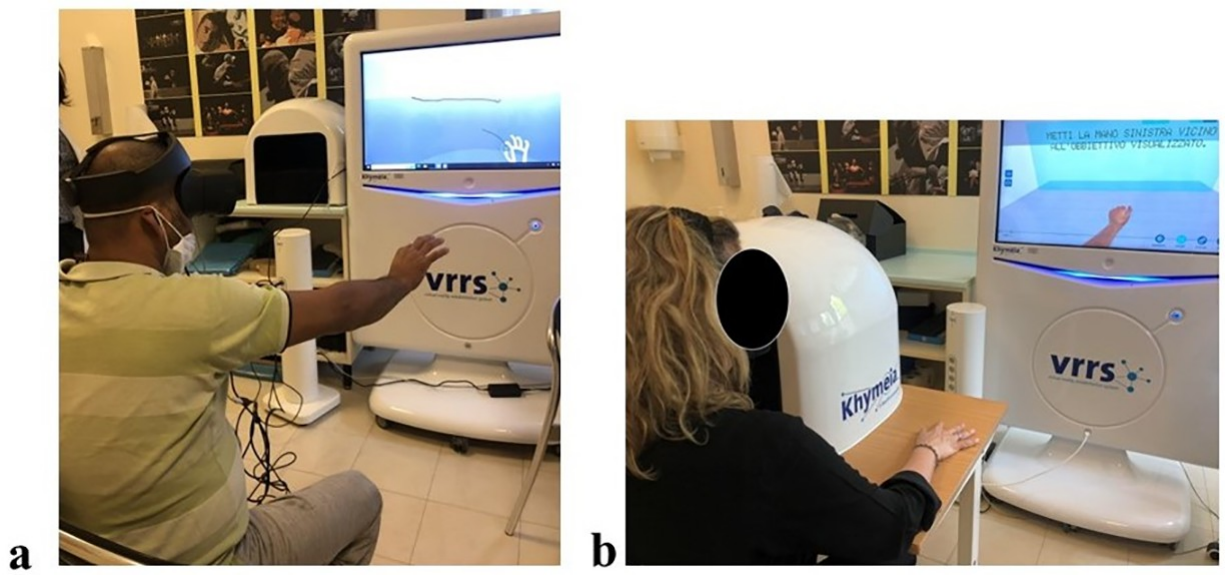
Experimental setting for VR interventions. (a) 3D—Virtual Reality session; (b) 2D Handbox session.

The VR protocol uses two types of technology: three-dimensional (3D) and two-dimensional (2D). During the 3D session, patient sits on a chair and wear the head-mounted display (HMD) while performing some exercises. The exergames consist of tasks requiring precision movements, such as building some blocks, putting the virtual block in a specific position, moving the virtual object inside the environment, paint and colouring some proper figures (Fig 2a). The 2D session is performed by the Virtual Reality Rehabilitation System-Handbox (Khymeia Group, Noventa Padovana, Italy) technology for hand and wrist rehabilitation that tracks the patient’s hand and project it to a monitor without the need to wear any sensor or HMD. Patient sits on a chair, and the monitor screen is positioned at a 1.5 m distance in front of the patient (Fig 2b). Through the Handbox, the patient executes hand exergames based on the pinch, grasp, single finger coordination, wrist movements, customized according to the patient’s motor ability. For both technologies, 3D and 2D, the arm and fingers are captured and projected onto the screen through the Leap Motion Controller. This advanced motion sensor device uses infrared cameras and LEDs to detect and track the position, orientation, and movement of each finger and the hand as a whole. It captures movements at a high frame rate, ensuring smooth and responsive interaction, reproducing an effect similar to the traditional mirror box [58]. For the 3D condition, the Leap Motion Controller is incorporated into the HMD, while for the 2D condition, it is integrated into the Handbox. Both systems allow patients to interact with virtual objects in a natural and intuitive manner. For example, patients can reach out, grab, move, and manipulate virtual objects as they would in the real world. During both sessions, 3D and 2D, there is the constant supervision of the physiotherapist and researcher supervision that guide the patients, directing their focus on the exercises and providing encouragement throughout the tasks.

#### 3.4.2. Experimental treatment

Participants of the experimental group will undergo treatment with Virtual Reality, both IVR and HB. The intervention will consist of 12 sessions lasting about 1 hour each and carried out with a frequency of three days per week within four weeks. Before starting the rehabilitation, arm illusion with the Handbox is inducted to explore the ability of the patients to perceive the virtual arm as their own. During this task, patients sit on a chair with the injured arm inside the Handbox and are invited to perform slow movements with the hand, such as moving the fingers one at a time and moving the wrist up and down. Moreover, they are invited to keep their attention to the virtual arm project on the screen that follows their natural movements. The arm illusion lasts 3 minutes, and the embodiment questionnaire is administered. Then, the treatment starts with IVR and HB sessions (Fig 2), which are counterbalanced to avoid the learning effect. During the IVR, patients sit on a chair and wear the HMD for the immersive experience while performing the abovementioned exercises. During the HB task, patients are sitting on a chair and invited to do some exergames projected on the Khymeia screen, such as inserting pegs into an abacus and pointing some targets, all with the injured arm. All tasks are administered in a complex sequence—the two IVR and HB sessions last half an hour each.

Furthermore, the therapist will establish the facilitation level for each exercise by fine-tuning the sensitivity of kinematic sensors, which range from 1 to 10 based on the patient’s motor impairment. This approach enables patients with minimal hand activity to successfully carry out the exercise. The personalization of exercises is determined by the patient’s baseline hand motor ability. Therefore, individuals with limited hand and arm capabilities will engage in exercises utilizing the Khymeia software’s high movement augmentation score, whereas those with more pronounced hand abilities will perform exercises with a lower augmentation facility score.

#### 3.4.3. Control treatment

Participants randomly assigned to the control group will receive rehabilitation treatment as per usual clinical practice. Specifically, patients will be directed to rehabilitation facilities according to standard clinical rehabilitation pathways. They will be assigned to a physiotherapist who will administer the rehabilitation treatment for the upper limb impairment. Patients in the control group will receive the same amount of rehabilitation for the recovery of upper limb impairment as the treatment group, which means 3 physiotherapy sessions lasting 1 hour 3 times a week for 4 weeks. Based on the treatment intensity, a physiotherapist ensured that the control group received the same level of treatment as the experimental group in terms of the physical effort required by patients.

### 3.5. Outcomes

In assessing the significance of outcome changes, several indicators are utilized. These include the *p*-value, which measures the probability that observed results are due to chance rather than the intervention itself. A *p*-value below a predetermined significance level, often 0.05 or 0.01, indicates statistical significance. Additionally, the confidence interval (CI) provides an estimate of the precision of treatment effects. A narrower CI suggests greater precision in estimating treatment effects. Finally, the Minimal Clinically Significant Difference (MCID) is utilized to assess the significant change perceived by patients regarding the treatment, evaluated through the satisfaction questionnaire.

#### 3.5.1. Primary outcome

The primary outcome measures will be the Fugl-Meyer for Upper Limbs [45] employed as a performance-based assessment to characterize motor recovery in research, has demonstrated outstanding reliability. This encompasses internal consistency, inter-rater reliability, intra-rater reliability, and test-retest reliability, particularly in the post-stroke context [59]. The assessment includes 33 items to evaluate upper extremity motor impairment and is scored between 0 and 2 (0 = unable, 1 = partly able, and 2 = fully able to complete movement) with a total score range of 0–66. The assessment will be performed before treatment (T1), after the conclusion of the treatment (T2), and after 6 months as a follow-up (T3).

#### 3.5.2. Secondary outcome

The secondary outcome will include timed tests that measure the improvement in the Box and Blocks Test [46] for the upper limb ability and motor coordination, MAIA test [47] and the Rubber Hand Illusion [48] for the patients’ proprioceptive ability; the Functional Independence Measure [55] and the Stroke Self-Efficacy [56] for the perceived and real ability in daily life activities. The assessment will be performed before treatment (T1), after the conclusion of the treatment (T2), and after 6 months as a follow-up (T3). Moreover, the satisfaction with the treatment received will also be assessed at T2.

#### 3.5.3. Sample size

A sample size of 30 participants per group has been determined using G*Power 3.1.3. This calculation was based on a repeated measures ANOVAs (within and between subjects), considering a small to medium effect size of 0.2 [60], as also reported in a two-arm Randomized Control Trial involving chronic stroke participants that also adopted as primary outcome measure the Fugl-Meyer scale [61]. The sample size determination also accounted for 20% attrition, with a significance level (α) set at 0.05 and a desired statistical power of 0.80, as outlined in the literature [62]. Additional, participants will be enlisted in the event of any dropouts.

#### 3.5.4. Recruitment and randomization

Patients considered eligible according to the inclusion criteria will be invited from the IRCCS Istituto delle Scienze Neurologiche di Bologna to participate in the study, explaining in detail the purposes and methods of the study. Before enrolment, informed consent will be obtained from patients. Then, to minimize the risk of bias, the Random Allocation Software 2.0 will be adopted to randomize participants and organize them into blocks of six, that is three participants receiving traditional rehabilitation and three participants receiving the Virtual Reality rehabilitation.

### 3.6. Data management

The participant center will send the case report form (CRF) to the data manager and the study’s principal investigator, and the file will be encrypted. Every patient will receive an alphanumeric identification code that prevents direct identification of the patient’s name. All data collected during the study will be stored and associated with this code. Only the data manager and authorized staff members can associate this code with the patient’s name. Once the data collection is terminated, they will be available from the following DOI: https://doi.org/10.6092/unibo/amsacta/7590.

### 3.7. Statistical analysis

The data analysis follows the Per Protocol approach, that is only participants who have adhered to the study protocol, patients who dropped out of the study are excluded from the analysis. Analysis will be performed through the Statistical Package for Social Sciences (SPSS). Descriptive statistics for both the experimental and control groups will encompass measures such as the mean and standard deviation. The student t-test for independent samples will be used to evaluate the baseline. In case of excessive deviation from normality, a similar non-parametric test (Mann-Whitney) will be used. Changes in motor performance and proprioception between T1 and the subsequent longitudinal evaluations (T2, T3) will be assessed using the repeated measures Analysis of Variance (rmANOVA) with a mixed design 2×3, considering the group as "between subjects" factor and the time point measure as "within subjects" factor. Residual plots will be inspected to verify linearity, normality and homoscedasticity assumptions for all models as well as to identify potential influential outliers. According to the literature, for all inferential analyses the probability of type 1 error is a-priori fixed at alpha α = 0.05 and will include reporting the 95% Confidence Interval for each estimate [62].

## 4. Discussion

The present protocol describes the background and the design of a study that aims to evaluate the effectiveness of a rehabilitation program with VR in improving sensorimotor and proprioception upper limb ability in patients with stroke. The central hypothesis is that the VR rehabilitation program could increase motor facilitation and, as a consequence, improve upper limb control and function compared to the control condition.

The present protocol was developed because motor impairments are the main consequence of stroke, together with the alteration in body representation and the sense of embodiment that prevent limb movements. Much literature has proven VR’s efficacy in motor rehabilitation after a stroke [26, 27]. However, less is known about the modulating role of the body ownership illusion in eliciting rehabilitation. In this line, a recent systematic review [34] demonstrated the significance of body illusion in promoting motor rehabilitation in patients with stroke, both for the upper [22, 63, 64] and lower limbs [23], compared to the control non-embodied condition. The enhanced feature of the embodied VR system, in contrast to conventional rehabilitation programs like the mirror box [58], lies in its ability to evoke motor imagery using computerized images. Additionally, the patient can receive remote feedback on their training, allowing them to ascertain the correctness of their rehabilitation tasks.

In the present protocol, the VR systems, IVR and HB, permit patients to see their real injured arm projected into the virtual environment, which is immersive for the IVR condition and augmented for the HB condition, stimulating the motor cortex [65, 66]. Moreover, thanks to the feature that allows the virtual arm to be adjusted according to the patient’s motor abilities, it is possible to generate an augmented movement even if the patient has minimal ability. Moreover, in contrast to traditional rehabilitation programs, VR treatments are typically well-received by patients due to their immersive environment and increased flexibility in catering to the patient’s clinical characteristics and progress. Additionally, patients have the opportunity to track and record their motor performance [30, 67].

The present protocol provides an integrated approach and requires a multi-professional team, from psychologists to physiotherapists and physiatrist, to plan a well-designed study with the possibility of correlating measures of functional outcomes and neuropsychological assessment.

To conclude, the induction of body ownership illusion in a VR rehabilitation program could be a step forward to traditional therapy that may enhance the upper limb motor recovery after a stroke, thus increasing the patients’ engagement with the treatment.

## 5. Limitations

Several limitations should be noted in the current clinical trial. Firstly, patient recruitment for stroke cases may prove challenging, given that the stroke unit at Bologna Hospital handles a relatively low number of cases. Secondly, the treatment’s duration—three days a week for four weeks—may pose a limitation, potentially impacting treatment adherence. Thirdly, patients, especially in the initial stages of stroke recovery, may experience clinical complications leading to treatment discontinuation or cessation. Lastly, we anticipate limitations on the technological front, such as technical issues or patients experiencing cybersickness, which could hinder their ability to complete the experimental sessions.

## Supporting information

S1 Checklist The CONSORT (Consolidated Standards of Reporting Trials) guidelines are a set of recommendations designed to improve the reporting of randomized controlled trials (RCTs). The primary goal of CONSORT is to ensure transparency, completeness, and clarity in the reporting of trial results, which in turn helps readers assess the validity and applicability of the findings.
(PDF)

S1 File Protocol for ethics committee-English version. (PDF)

S2 File Protocol for ethics committee-Italian version. (PDF)

S3 File (DOCX)
