# Correction: Effectiveness of a virtual reality rehabilitation in stroke patients with sensory-motor and proprioception upper limb deficit: A study protocol

**DOI:** 10.1371/journal.pone.0321332

**Published:** 2025-03-31

**Authors:** Sara Ventura, Alessia Tessari, Sara Castaldini, Elisabetta Magni, Andrea Turolla, Rosa Baños, Giada Lullini

There is an error in affiliation 3 for authors Sara Castaldini, Elisabetta Magni and Giada Lullini. The correct affiliation 3 is: UOC Medicina Riabilitativa e Neuroriabilitazione, IRCCS Istituto delle Scienze Neurologiche di Bologna, Bologna, Italy.

The funding statement for this article is incorrect. The correct funding statement is as follows: This work was supported by the Margarita Salas postdoctoral fellowship from the Ministry of Universities of the Government of Spain (European Union NextGeneration EU, Ref. UP2021-044). The publication of this article was funded by the “Ricerca Corrente” program of the Italian Ministry of Health. The funders had no role in the study design, data collection and analysis, decision to publish, or preparation of the manuscript.
