# Supplementary material for: Effectiveness of a Virtual Reality rehabilitation in stroke patients with sensory-motor and proprioception upper limb deficit: A study protocol
Source: PLoS One. 2024 Aug 12;19(8):e0307408. doi: 10.1371/journal.pone.0307408 (PMC11318908; doi:10.1371/journal.pone.0307408)
Supplement: S1 File — (PDF) [file pone.0307408.s002.pdf]

STUDY PROTOCOL

**Title**	Effectiveness of virtual reality rehabilitation in stroke patients with sensory-motor and proprioception upper limb deficit: a pilot study
**Acronymous**	VR-STROKE
**Code**	ISNB-MR&NR-2022-03
**Place**	UO Medicina Riabilitativa e Neuroriabilitazione (SC) IRCCS ISNB, Ausl di Bologna
**Principal investigator**	Giada Lullini
**Promotor**	IRCCS ISNB, Ausl di Bologna
**Financial entity**	NA

Authors:, Giada Lullini[1], Sara Castaldini[1], Alessia Tessari[2] , Sara Ventura[2], Annalisa Di Gioia[1], Elisabetta Magni[1], Andrea Turolla[3]

[1]UOC di Medicina Riabilitativa e Neuro-riabilitazione, IRCCS Istituto delle Scienze Neurologiche di Bologna, Azienda USL Bologna

[2]Dipartimento di Psicologia dell'Università degli Studi di Bologna

[3] Dipartimento di scienze Biomediche e Neuromotorie dell'Università degli Studi di Bologna

Rols and responsabilities:

The experimental center are:

- UO di Medicina Riabilitativa e Neuro-riabilitazione (SC) dell'IRCCS Istituto delle Scienze Neurologiche

The collaborative centers are:

- University of Bologna, Department of Psychology
- University of Bologna, Department of Biomedical Science

1. Introduction

According to European Cardiovascular Disease Statistics, stroke is the second leading cause of death in Europe, accounting for an average of 405.000 deaths (9%) in men and 583.000 deaths (13%) in women (Eurostat Statistics Explained, 2022). This prevalence is reflected in Italy, where stroke is responsible for 9-10% of all deaths and represent the leading cause of disability. Each year, approximately 90.000 stroke-related hospitalizations are recorded in Italy, with 20% being recurrent incidents (Ministry of Health, 2022).

Stroke incidence increases after the age of 55, doubling with each subsequent decade, and 75% of cases occur in individuals over 65. Following a stroke, 20-30% of individuals die within a month, and 40-50% within the first year. Of those who survive, only 25% achieve complete recovery, while the remaining 75% undergo a rehabilitation phase lasting from weeks to months or even years. At the end of this period, patients may experience full recovery or remain in a state of disability throughout their lives (Langhorne et al., 2009).

Among the primary disabilities resulting from stroke is motor impairment of the upper limbs, leading to partial or complete inability to move the affected limb. This impacts the ability to perform reaching tasks due to impaired motor coordination or an inability to control grip and finger strength during object manipulation (Steenbergen & Van Der Kamp, 2004). In daily life, this disability manifests as an inability to eat, dress, or attend to personal hygiene independently, resulting in dependence on a caregiver and negatively affecting the quality of life. Therefore, continuous rehabilitation to regain motor skills is vital for both the patient and their caregivers (Duncan et al., 1994).

From a neuroscientific perspective, movement planning, preparation, and execution deficits can result from damage to primary motor cortix area responsible for praxis control, or distorted body representation, altering proprioceptive and kinesthetic signals and the perception of peripersonal space (Wallwork et al., 2016). Consequently, patients may experience an altered the sense of embodiment, or the perception of their own body. Specifically, the sense of embodiment consists of three factors: a) ownership, the perception of possessing one's own body; b) agency, the perception of motor control over one's body; and c) location, the perception of being physically in the same place as the body. Together, these factors allow individuals to perceive, act, and interact in their surrounding environment (Kilteni et al., 2012).

Recent studies have shown that intervening through the illusion of corporeal self in hemiplegic patients improves the rehabilitation of the affected limb (Cha et al., 2021; Tambone et al., 2021). One of the systems used for generating the illusion of the limb is the mirror box, a tool in which the patient

embodies the healthy arm reflected in a mirror. If the illusion is successful, the patient perceives the reflected arm in the mirror as their own instead of the impaired limb (Ezendam et al., 2009). This may be possible because the embodiment of the reflected hand improves the reorganization of body representation in post-stroke patients with motor deficits (Tosi et al., 2018). Rehabilitation through limb illusion has been further translated into virtual reality, demonstrating its effectiveness in upper limb rehabilitation (Weber et al., 2019; Hsu et al., 2022).

In recent decades, virtual reality has been widely used in upper limb motor rehabilitation following a stroke, yielding significant results when compared to traditional rehabilitation programs (Holden et al., 2007; Kim et al., 2020). Virtual reality is an advanced technology that provides interactive environments replicating the surrounding reality. It is divided into immersive and non-immersive virtual reality; the former allows the patient to feel present in the virtual environment through a head-mounted display, while the latter projects the virtual environment onto a screen, and the patient interacts with it through devices such as joysticks or cybergloves (Henderson et al., 2007). In rehabilitation, virtual reality improves neuroplasticity and motor recovery by providing engaging training with real-time feedback, customization of exercises based on cognitive and motor abilities, immersive and interactive experiences, and faithful simulation of real-world activities (Turolla et al., 2013). However, the effectiveness of incorporating the illusion of one's limb into virtual reality rehabilitation is not yet fully understood.

Therefore, given the high incidence of stroke, the resulting disabilities, and the need for prompt and continuous rehabilitation, along with promising data on virtual reality as an effective tool for sensorimotor and proprioceptive rehabilitation, this project aims to study the efficacy of a virtual reality system for upper limb rehabilitation using the corporeal self-illusion paradigm.

1.1. Study hypotheses

This study is based on the hypothesis that a neurorehabilitation protocol utilizing virtual reality will be effective in the sensorimotor and proprioceptive rehabilitation of the upper limb in patients with post-stroke outcomes. Additionally, it is hypothesized that administering a rehabilitation protocol with virtual reality may prove more effective than the traditional rehabilitation protocol administered in routine clinical practice for recovering sensorimotor and proprioceptive deficits in the upper limb of patients with post-stroke outcomes.

1.2. Study aim

The primary aim of this study is to assess the effectiveness of a neurorehabilitation protocol using a virtual reality system for recovering sensorimotor and proprioceptive deficits in the upper limb of patients with post-stroke outcomes.

The secondary objective is to compare the outcomes, defined as the recovery of sensorimotor and proprioceptive function in the upper limb, between patients undergoing rehabilitation with virtual reality and those undergoing standard upper limb rehabilitation as per routine clinical practice.

2. Study objectives

2.1. Primary abjective

Assessing the effect of a rehabilitation protocol using a virtual reality system for the recovery of sensorimotor and proprioceptive function in the upper limb in patients with stroke outcomes.

2.2. Secondary objective

- Comparing the outcomes of a rehabilitation protocol using virtual reality against the standard rehabilitation protocol administered as per normal clinical practice for the recovery of sensorimotor and proprioceptive function in the upper limb in patients with stroke outcomes.
- Assessing the satisfaction of patients with stroke outcomes regarding the implementation of an upper limb rehabilitation program conducted with virtual reality.

3. Material and method

3.1. Study design

Randomized control trial without pharmaceuticals use.

3.2. Population

Inclusion criteria:

- Age between 18 and 85 years;
- Female and male;
- Diagnosis of stroke outcomes occurring between 2 months and 18 months prior to enrollment;
- Impairment of upper limb functionality (Motricity Index with total score for the upper limb ≤ 80);
- Sensorimotor and proprioceptive impairment of the upper limb (Negative Thumb Location Test in 3 out of 4 trials);
- Signed informed consent to participate in the study.

Exclusion criteria:

- Ongoing clinical instability;

- Severe psychiatric comorbidity that may interfere with adherence to the proposed treatment protocol (e.g., severe personality disorders, severe psychomotor agitation);
- Severe impairments in upper limb motor function: Motricity Index Scale with the following cutoffs: pinch grip < 11, elbow flexion < 14, shoulder abduction < 14;
- Severe deficit in verbal comprehension: Token Test (Spinnler 1987) with a score < 2;
- Severe spatial neglect: Barrage Test (Albert 1973) score > 3.

3.3. Procedure

Patients who meet the inclusion criteria, in the absence of all exclusion criteria, will be recruited at the Department of Rehabilitation Medicine and Neuro-rehabilitation (SC) of IRCCS Institute of Neurological Sciences in Bologna after signing the informed consent. For recruitment purposes, the Principal Investigator (PI) or a delegate will explain to the patient the rationale and operational procedures of the study using the information provided in the study information sheet. After enrollment, the included patients will be randomized into two groups: a treatment group (VR) that will undergo upper limb rehabilitation for proprioceptive and sensorimotor recovery using the Khymeia system with virtual reality exercises, and a control group (C) that will undergo upper limb rehabilitation according to standard clinical practice. Both groups will receive the same number of rehabilitation sessions with the same duration and weekly frequency for the recovery of sensorimotor and proprioceptive function in the upper limb. If included patients need to undergo a rehabilitation protocol for the recovery of other deficits related to the stroke event (e.g., language disorders, dysphagia, hemiplegia of the lower limbs, gait abnormalities), the execution of the rehabilitation pathway will be ensured according to standard clinical practice for those disorders.

The randomization list will be generated by the PI before the start of the study using Microsoft Excel and kept by an investigator not involved in patient assessment. Patients will be assigned to the two groups following the randomization sequence. Subsequently, patients in both groups will undergo pre-test evaluations (T0).

3.3.1. Experimental group (virtual reality)

The rehabilitation team (comprising a physician, speech therapist, physiotherapist, and psychologist) will establish an individualized rehabilitation protocol for the recovery of sensorimotor and proprioceptive function in the upper limb. This protocol will involve exergaming exercises with virtual reality, totaling 50 minutes, conducted three days a week for four consecutive weeks. The rehabilitation protocol utilizing virtual reality will be carried out using the VRRS Khymeia system. This system will include exercises in semi-immersive virtual reality (utilizing the VRRS HandBox,

an advanced upper limb skeleton tracking device) and immersive virtual reality exercises (created with a Head Mounted Display device).

Rehabilitation sessions will take place at the Department of Rehabilitation Medicine and Neuro-rehabilitation at the IRCCS Institute of Neurological Sciences in Bologna.

3.3.2. Control group

Patients in the control group will undergo rehabilitation treatment for sensorimotor and proprioceptive deficits in the upper limb according to standard clinical practice. They will have sessions three times a week, each lasting 50 minutes, for a duration of 4 weeks.

At the end of the 4-week treatment period, patients from both the treatment (VR) and control (C) groups will undergo post-test evaluations (T1).

3.3.3. Pre-Treatment assessment

a) Motricity Index Test (Bohannon, 1999)
b) Fugl-Meyer Test (Fugl-Meyer et al., 1975)
c) Box and Block Test (Mathiowetz et al., 1985)
d) Functional Indipendence Measure (FIM) (Beninato et al., 2006)
e) Multidimensional Assessment of Interoceptive Awareness (MAIA) (Machorrinho et al., 2019)
f) Thumb Location Test (Rand, 2018)
g) Rubber Hand Illusion test (Romano et al., 2021)
h) STIMA test (Tessari et al., 2015)
i) Intelligence: Raven Progressive Matrices PM47 (Carlesimo et al., 1996);
j) Executive functions: Trail Making Test, parte A (attenzione) e B (Giovagnoli et al., 1996);
k) Memory: 15 words memory test (Ray, 1994), Corsi spatial short-term memory (Spinnler, 1987), digit span for verbal short-term memory digit (Orsini et al., 1987);
l) Visuo-spatial ability: VOSP, Visual Object and Space Perception battery; shape detection and shape decision (Warrington & James, 1991)
m) Attention: trial making test, matrici attentive, fluenza alternata.

3.3.4. Post-Treatment Assessment

a) Motricity Index Test (Bohannon, 1999)
b) Fugl-Meyer Test (Fugl-Meyer et al., 1975)
c) Box and Block Test (Mathiowetz et al., 1985)
d) Functional Indipendence Measure (FIM) (Beninato et al., 2006)
e) Multidimensional Assessment of Interoceptive Awareness (MAIA) (Machorrinho et al., 2019)

f) Thumb Location Test (Rand, 2018) NO
g) Rubber Hand Illusion test (Romano et al., 2021)
h) STIMA test (Tessari et al., 2015) NO
i) Attention: trial making test, matrici attentive, fluenza alternata.
j) Client Satisfaction Questionnaire-8

4. Outcome

4.1. Primary endpoint

In order to assess the effect that the proposed treatment has on the sensorimotor and proprioceptive impairment of the upper limb, a comparison is conducted between the baseline assessments (T0) and those performed at the end of the 4-week rehabilitation treatment (T1).

4.2. Secondary endpoint

- To compare the effect of virtual reality rehabilitation for sensorimotor and proprioceptive deficits in the upper limb to conventional rehabilitation, a comparison is conducted between the assessments at T0 and T1 within the virtual reality treatment group (VR) and the group undergoing rehabilitation as per standard clinical practice (C).
- To assess patient satisfaction with the proposed experimental treatment, a validated questionnaire is administered to investigate patient satisfaction. The data are processed by calculating the mean, median, mode, and standard deviation for all 8 items. This approach allows for identifying the patient's overall considerations regarding the proposed treatment through a statistical summary that includes measures of central tendency (mean, median, mode) and data dispersion (standard deviation).

5. Data analysis

5.1. Sample size

This study plans to enroll 20 patients, with 10 in the experimental group (VR) and 10 in the control group (C). Given that the study duration is 24 months, with the last 6 months dedicated to statistical analysis, results analysis, and scientific dissemination, the active recruitment and patient inclusion period is estimated to be 18 months. Each patient will participate in the study for 6 weeks (1 week for the baseline assessment at T0, 4 weeks for rehabilitation, and 1 week for the post-treatment assessment at T1). It is believed that the overall number of 20 included patients is achievable within the study's timeframe.
As this is a pilot study, the sample size is considered sufficient to achieve the set objectives, taking into account a potential dropout rate of 20%.

5.2. Statistical analysis

The data analysis will be conducted using the Statistical Package for Social Science (SPSS version 26.0).

The preliminary data obtained from the pilot study will allow for estimating the variation in outcome measures between pre-test and post-test and between the treatment and control groups, providing insights for a potential subsequent trial with a larger sample.

The data will be analyzed based on the stated objectives, which include:

- Descriptive analysis of socio-demographic data for the experimental and control groups, including mean and standard deviation.
- The effect of the virtual reality rehabilitation protocol in the treated group will be assessed by comparing the scores of evaluation scales at pre-test and post-test. Data distribution will be tested using the Shapiro-Wilk test. The non-parametric Friedman test will be used in case of non-normal distribution, while ANOVA will be utilized in case of normal distribution. The significance level will be defined at $p \leq 0.05$.
- The effect of the virtual reality rehabilitation protocol compared to the standard rehabilitation protocol will be assessed by comparing the scores of scales at T0 and T1 between the VR and C groups using the non-parametric Mann-Whitney test for independent samples.

6. Relevance

If the experimental hypotheses will be confirmed, the use of the virtual reality rehabilitation system could be implemented in routine clinical practice to rehabilitate sensorimotor integration in patients with stroke outcomes. An innovative approach employing virtual reality techniques aims not only to enhance patient treatment adherence but also to support proprioceptive and sensorimotor rehabilitation through the body illusion paradigm, which is not achievable in traditional rehabilitation. The enhancement of proprioceptive and sensorimotor aspects is considered crucial for the rehabilitation of neuromotor impairment. Therefore, by promptly implementing this type of intervention following a cerebrovascular event, one could hope for a faster and more effective functional recovery.

7. Feasibility

- The Rehabilitation Medicine and Neuro-rehabilitation Unit of the IRCCS Institute of Neurological Sciences in Bologna coordinates both the acute rehabilitation phase (with daily consultations at the Stroke Unit) and the sub-acute phase of the Stroke Clinical Pathway. Consequently, they daily admit patients with stroke outcomes at various stages of the

rehabilitation process. These patients may possess the characteristics to be included in the project.

- The Rehabilitation Medicine and Neuro-rehabilitation Unit of the IRCCS Institute of Neurological Sciences in Bologna is equipped with the VRRS KHYMEIA rehabilitation system.
- The Department of Psychology at the University of Bologna has the expertise to assess the neurocognitive aspects of enrolled patients and administer relevant evaluation scales.
- The Department of Biomedical and Neuromotor Sciences at the University of Bologna has the expertise to assess the neuromotor and proprioceptive aspects of enrolled patients.

8. Ethical aspects

8.1. Informed consent

Each potential participant will be provided with a comprehensive explanation of the study procedures, and they will have the opportunity to ask questions and receive answers to any doubts they may have. The informed consent form must be signed by the participant or by a legally authorized representative before their participation in any of the study procedures. The documentation pertaining to the patient must demonstrate that consent was obtained before participation in the study. A copy of the signed informed consent form must be provided to the patient, and the original signed form must be retained at the study site.

8.2. Approval of ethical committee

The study will be conducted in accordance with international standards ISO 14:155, adhering to Good Clinical Practice and in compliance with the ethical principles outlined in the Declaration of Helsinki (64th WMA General Assembly, Fortaleza, Brazil, 2013). This protocol, the informed consent form, and all relevant information related to the study will be submitted to the appropriate Ethics Committee for evaluation and approval before the study commences. The current protocol poses no health risks to the participants. Any modifications to the protocol will be proposed to the Local Ethics Committee as an amendment request.

8.3. Data policy

Adequate documentation will be maintained for all aspects related to the patient's clinical data, worksheets, nursing notes, adverse event notification forms, and study discontinuation forms. Personal data will be stored in compliance with GDPR guidelines. The results will be presented exclusively in aggregated and anonymized form.

9. Dissemination

The principal investigator commits to producing the final report, publishing all collected data as described in the protocol, and ensuring that the data are reported responsibly and consistently. In particular, the publication of data resulting from this study will occur irrespective of the obtained results. The transmission or dissemination of data through scientific publications and/or presentations at conferences, congresses, and seminars will only occur following the purely statistical analysis of the data or, in any case, in a completely anonymous form.

References

M. della salute. (2022.). *No Title*. www.salute.gov.it

Beninato, M., Gill-Body, K. M., Salles, S., Stark, P. C., Black-Schaffer, R. M., & Stein, J. (2006). Determination of the minimal clinically important difference in the FIM instrument in patients with stroke. *Archives of Physical Medicine and Rehabilitation*, *87*(1), 32–39.

Bohannon, R. W. (1999). Motricity index scores are valid indicators of paretic upper extremity strength following stroke. *Journal of Physical Therapy Science*, *11*(2), 59–61.

Cha, K., Wang, J., Li, Y., Shen, L., Chen, Z., & Long, J. (2021). A novel upper-limb tracking system in a virtual environment for stroke rehabilitation. *Journal of Neuroengineering and Rehabilitation*, *18*, 1–13. https://doi.org/http://dx.doi.org/10.1186/s12984-021-00957-6

Duncan, P. W., Goldstein, L. B., Horner, R. D., Landsman, P. B., Samsa, G. P., & Matchar, D. B. (1994). Similar motor recovery of upper and lower extremities after stroke. *Stroke*, *25*(6), 1181–1188. https://doi.org/10.1161/01.STR.25.6.1181

Explained, E. S. (2022). (n.d.). *No Title*. https://ec.europa.eu/eurostat/statistics-explained/index.php?title=Cardiovascular_diseases_statistics

Ezendam, D., Bongers, R. M., & Jannink, M. J. A. (2009). Systematic review of the effectiveness of mirror therapy in upper extremity function. *Disability and Rehabilitation*, *31*(26), 2135–2149. https://doi.org/10.3109/09638280902887768

Fugl-Meyer, A. R., Jääskö, L., Leyman, I., Olsson, S., & Steglind, S. (1975). A method for evaluation of physical performance. *Scand J Rehabil Med*, *7*, 13–31.

Henderson, A., Korner-Bitensky, N., & Levin, M. (2007). Virtual reality in stroke rehabilitation: a systematic review of its effectiveness for upper limb motor recovery. *Topics in Stroke Rehabilitation*, *14*(2), 52–61.

Holden, M. K., Dyar, T. A., & Dayan-Cimadoro, L. (2007). Telerehabilitation using a virtual environment improves upper extremity function in patients with stroke. *IEEE Transactions on Neural Systems and Rehabilitation Engineering*, *15*(1), 36–42.

Hsu, H.-Y., Kuo, L.-C., Lin, Y.-C., Su, F.-C., Yang, T.-H., & Lin, C.-W. (2022). Effects of a Virtual Reality–Based Mirror Therapy Program on Improving Sensorimotor Function of Hands in Chronic Stroke Patients: A Randomized Controlled Trial. *Neurorehabilitation and Neural Repair*, 15459683221081430.

Kilteni, K., Groten, R., & Slater, M. (2012). The Sense of Embodiment in Virtual Reality. *PRESENCE-TELEOPERATORS AND VIRTUAL ENVIRONMENTS*, *21*(4), 373–387. https://doi.org/10.1162/PRES_a_00124 WE - Science Citation Index Expanded (SCI-EXPANDED) WE - Social Science Citation Index (SSCI)

Kim, W.-S., Cho, S., Ku, J., Kim, Y., Lee, K., Hwang, H.-J., & Paik, N.-J. (2020). Clinical application of virtual reality for upper limb motor rehabilitation in stroke: review of technologies and clinical evidence. *Journal of Clinical Medicine*, *9*(10), 3369.

Langhorne, P., Coupar, F., & Pollock, A. (2009). Motor recovery after stroke: a systematic review. *The Lancet Neurology*, *8*(8), 741–754.

Machorrinho, J., Veiga, G., Fernandes, J., Mehling, W., & Marmeleira, J. (2019). Multidimensional assessment of interoceptive awareness: Psychometric properties of the Portuguese version. *Perceptual and Motor Skills*, *126*(1), 87–105.

Mathiowetz, V., Volland, G., Kashman, N., & Weber, K. (1985). Adult norms for the Box and Block Test of manual dexterity. *The American Journal of Occupational Therapy*, *39*(6), 386–391.

Pollock, A., Farmer, S. E., Brady, M. C., Langhorne, P., Mead, G. E., Mehrholz, J., & van Wijck, F. (2014). Interventions for improving upper limb function after stroke. *Cochrane Database of Systematic Reviews*, *11*.

Rand, D. (2018). Proprioception deficits in chronic stroke—Upper extremity function and daily living. *PloS One*, *13*(3), e0195043.

Romano, D., Maravita, A., & Perugini, M. (2021). Psychometric properties of the embodiment scale for the rubber hand illusion and its relation with individual differences. *SCIENTIFIC REPORTS*, *11*(1). https://doi.org/10.1038/s41598-021-84595-x WE - Science Citation Index Expanded (SCI-EXPANDED) WE - Social Science Citation Index (SSCI)

Steenbergen, B., & Van Der Kamp, J. (2004). Control of prehension in hemiparetic cerebral palsy: similarities and differences between the ipsi-and contra-lesional sides of the body. *Developmental Medicine and Child Neurology*, *46*(5), 325–332.

Tambone, R., Giachero, A., Calati, M., Molo, M. T., Burin, D., Pyasik, M., Cabria, F., & Pia, L. (2021). Using Body Ownership to Modulate the Motor System in Stroke Patients. *Psychological Science*, *32*(5), 655–667. https://doi.org/10.1177/0956797620975774

Tessari, A., Toraldo, A., Lunardelli, A., Zadini, A., & Rumiati, R. I. (2015). STIMA: a short screening test for ideo-motor apraxia, selective for action meaning and bodily district. *Neurological Sciences*, *36*(6), 977–984.

Tosi, G., Romano, D., & Maravita, A. (2018). Mirror box training in hemiplegic stroke patients affects body representation. *Frontiers in Human Neuroscience*, *11*, 617.

Turolla, A., Dam, M., Ventura, L., Tonin, P., Agostini, M., Zucconi, C., Kiper, P., Cagnin, A., & Piron, L. (2013). Virtual reality for the rehabilitation of the upper limb motor function after stroke: a prospective controlled trial. *Journal of Neuroengineering and Rehabilitation*, *10*(1), 1–9.

Wallwork, S. B., Bellan, V., Catley, M. J., & Moseley, G. L. (2016). Neural representations and the cortical body matrix: implications for sports medicine and future directions. *British Journal of Sports Medicine*, *50*(16), 990–996.

Weber, L. M., Nilsen, D. M., Gillen, G., Yoon, J., & Stein, J. (2019). Immersive virtual reality mirror therapy for upper limb recovery following stroke: A pilot study. *American Journal of Physical Medicine & Rehabilitation*, *98*(9), 783.
