# Supplementary material for: Effectiveness of a Virtual Reality rehabilitation in stroke patients with sensory-motor and proprioception upper limb deficit: A study protocol
Source: PLoS One. 2024 Aug 12;19(8):e0307408. doi: 10.1371/journal.pone.0307408 (PMC11318908; doi:10.1371/journal.pone.0307408)
Supplement: S2 File — (PDF) [file pone.0307408.s003.pdf]

PROTOCOLLO DI STUDIO

**Titolo dello studio**	Efficacia di un protocollo riabilitativo in realtà virtuale in pazienti con esiti di stroke e deficit senso-motorio e propriocettivo dell'arto superiore: studio pilota
**Acronimo dello studio**	VR-STROKE
**Codice del protocollo**	ISNB-MR&NR-2022-03
**Struttura Coinvolta**	UO Medicina Riabilitativa e Neuroriabilitazione (SC) IRCCS ISNB, Ausl di Bologna
**Principal investigator**	Giada Lullini
**Promotore**	IRCCS ISNB, Ausl di Bologna
**Finanziatore**	NA

Autori del Protocollo: Giada Lullini[1], Sara Castaldini[1], Alessia Tessari[2], Sara Ventura[2], Annalisa Di Gioia[1], Elisabetta Magni[1], Andrea Turolla[3]

[1]UOC di Medicina Riabilitativa e Neuro-riabilitazione, IRCCS Istituto delle Scienze Neurologiche di Bologna, Azienda USL Bologna

[2]Dipartimento di Psicologia dell'Università degli Studi di Bologna

[3] Dipartimento di scienze Biomediche e Neuromotorie dell'Università degli Studi di Bologna

Ruoli e responsabilità:

Il centro sperimentatore è l'UO di Medicina Riabilitativa e Neuro-riabilitazione (SC) dell'IRCCS Istituto delle Scienze Neurologiche

Il Dipartimento di Psicologia UNIBO collabora allo studio

Il Dipartimento di Scienze Biomediche e Neuromotorie UNIBO collabora allo studio

1. Introduzione

Secondo l'European Cardiovascular Disease Statistics l'ictus è la seconda causa di morte in Europa, con una media di 405.000 decessi (9%) negli uomini e 583.000 (13%) decessi nelle donne (Eurostat Statistics Explained, 2022). La percentuale si riflette anche in Italia in cui l'ictus è responsabile del 9-10% di tutti i decessi e rappresenta la prima causa di invalidità. Secondo i dati Istat, ogni anno si registrano in Italia circa 90.000 ricoveri dovuti ad un ictus cerebrale, di cui il 20% sono recidive (Mistero della salute, 2022). L'ictus è più frequente dopo i 55 anni, la sua prevalenza raddoppia successivamente ad ogni decade e il 75% si verifica nelle persone con più di 65 anni. A seguito di un ictus, il 20-30% delle persone muore entro un mese dall'evento e il 40-50% entro il primo anno. In caso di sopravvivenza, solo il 25% dei pazienti guarisce completamente, mentre il restante 75% dei sopravvissuti andrà incontro ad una fase di riabilitazione, di durata variabile, da alcune settimane ad alcuni mesi, o anni. Alla fine di questo periodo il paziente può sperimentare un pieno recupero o rimanere in uno stato di disabilità per tutta la vita (Langhorne et al., 2009).

Tra le principali disabilità dovute ad un ictus troviamo la disabilità motoria degli arti superiori che comporta una parziale o completa incapacità di muovere l'arto destro o sinistro a seconda dell'emisfero danneggiato (Pollock et al., 2014). In particolare, il paziente riscontra difficoltà nell'eseguire compiti di raggiungimento dovuti alla mancata coordinazione motoria o incapacità di controllare la presa e la forza delle dita durante la manipolazione degli oggetti (Steenbergen & Van Der Kamp, 2004). Nella vita quotidiana, tale disabilità si riflette nell'incapacità di mangiare da soli, vestirsi, provvedere privatamente alla propria igiene personale, con la conseguente dipendenza da un caregiver, impattando negativamente sulla qualità della vita. Pertanto, una costante riabilitazione per riacquisire le abilità motorie risulta vitale per il paziente e per i suoi cari (Duncan et al., 1994).

Dal punto di vista delle neuroscienze, il deficit nella pianificazione, preparazione ed esecuzione del movimento può risultare da un danno alle cortecce motorie primarie o alle regioni preposte al controllo prassico o alle rappresentazioni corporee distorte, che alterano i segnali propriocettivi e cinestesici, e la percezione dello spazio peripersonale (Wallwork et al., 2016). Di conseguenza, il paziente presenta un'alterazione nel senso di embodiment, o la percezione del sé corporeo. Nello specifico, il sé corporeo è composto da tre fattori: *proprietà* intesa come la percezione di possedere il proprio corpo, *agenza* intesa come la percezione di avere il controllo motorio sul proprio corpo, *locazione* intesa come la percezione di essere

fisicamente nello stesso posto in cui è il corpo. Tali fattori, nel loro insieme, permettono all'individuo di percepirsi e di agire e interagire nell'ambiente circostante (Kilteni et al., 2012). Studi recenti hanno dimostrato che intervenire attraverso l'illusione del sé corporeo in pazienti emiplegici migliora la riabilitazione dell'arto leso (Cha et al., 2021; Tambone et al., 2021). Uno dei sistemi utilizzati in letteratura per generare l'illusione dell'arto è la mirror box, uno strumento in cui il paziente incarna il braccio sano riflesso allo specchio che, se l'illusione è raggiunta, permette al paziente di percepire come proprio il braccio riflesso nello specchio al posto del suo braccio leso (Ezendam et al., 2009). Questo potrebbe essere possibile perché l'incarnazione della mano riflessa dallo specchio migliorerebbe la riorganizzazione della rappresentazione del corpo in pazienti con deficit motorio post ictus (Tosi et al., 2018). La riabilitazione mediante l'illusione del braccio è stata in seguito traslata in realtà virtuale, dimostrando la sua efficacia nella riabilitazione dell'arto superiore (Weber et al., 2019; Hsu et al., 2022).

Negli ultimi decenni, infatti, la realtà virtuale è stata ampiamente utilizzata nella riabilitazione motoria dell'arto superiore in seguito a un ictus, apportando risultati significati quando comparata con un programma di riabilitazione tradizionale (Holden et al., 2007; Kim et al., 2020). La realtà virtuale è una tecnologia avanzata che fornisce ambienti interattivi che riproducono la realtà circostante. Essa si divide in realtà virtuale immersiva e non immersiva; la prima permette al paziente di sentirsi presente nell'ambiente virtuale grazie a un caschetto chiamato *head mounted display* che proietta l'ambiente tridimensionale; la seconda, invece, proietta l'ambiente virtuale su di uno schermo e il paziente può interagirci attraverso dei dispositivi quali *joystick* o *cyberglove* (Henderson et al., 2007). Nel campo della riabilitazione, la realtà virtuale è in grado di migliorare la neuroplasticità e il recupero motorio in quanto fornisce al paziente un training coinvolgente grazie a diversi aspetti quali i feedback in tempo reale, la customizzazione degli esercizi in base alle abilità cognitive e motorie del paziente, l'esperienza immersiva e interattiva che la tecnologia offre, e una fedele simulazione delle attività del mondo reale (Turolla et al., 2013). Tuttavia, la realtà virtuale si è dimostrata efficace nella riabilitazione dell'arto superiore, ma si sa ancora poco di quanto l'integrazione dell'illusione del proprio arto possa portare beneficio alla riabilitazione.

Pertanto, considerando la forte incidenza dell'ictus, la disabilità che comporta e la necessità di una tempestiva e costante riabilitazione, insieme ai dati promettenti della realtà virtuale come strumento efficace per la riabilitazione sensomotoria e propriocettiva, il

presente progetto ha l'obiettivo di studiare l'efficacia di un sistema di realtà virtuale per la riabilitazione dell'arto superiore attraverso il paradigma dell'illusione del sé corporeo.

1.1. Ipotesi sperimentali

Questo studio si basa sull'ipotesi che un protocollo di neuro-riabilitazione basato su realtà virtuale risulti efficace nella riabilitazione senso-motoria e propriocettiva dell'arto superiore in pazienti con esiti di stroke. Inoltre si ipotizza che la somministrazione di un protocollo riabilitativo con realtà virtuale possa essere più efficace rispetto al protocollo riabilitativo tradizionale somministrato nella normale pratica clinica nel recupero dei deficit senso-motori e propriocettivi dell'arto superiore nei pazienti con esiti di stroke.

1.2. Scopo dello studio

Lo scopo dello studio è quello di valutare l'effetto di un protocollo di neuro-riabilitazione con sistema a realtà virtuale per il recupero del deficit senso-motorio e propriocettivo dell'arto superiore in pazienti con esiti di stroke.

Lo scopo secondario è confrontare l'outcome (inteso come recupero della funzione senso-motoria e propriocettiva dell'arto superiore) nei pazienti sottoposti a riabilitazione con realtà virtuale rispetto a pazienti che svolgono riabilitazione standard dell'arto superiore come da normale pratica clinica.

2. Obiettivi dello studio

2.1. Obiettivo primario:

Valutare l'effetto di un protocollo di riabilitazione con sistema a realtà virtuale per il recupero della funzione senso-motoria e propriocettiva dell'arto superiore in pazienti con esiti di stroke.

2.2. Obiettivi secondari:

- Confrontare l'outcome di un protocollo riabilitativo con realtà virtuale rispetto al protocollo riabilitativo standard somministrato come da normale pratica clinica per il recupero senso-motorio e propriocettivo dell'arto superiore in pazienti con esiti di stroke.

- Valutare la soddisfazione dei pazienti con esiti di stroke rispetto allo svolgimento di un programma riabilitativo dell'arto superiore svolto con realtà virtuale

3. Materiali e metodi

Studio pilota interventistico senza dispositivi e senza farmaci.

3.1. Popolazione:

Criteri di inclusione:

1) Età compresa tra i 18 e i 85 anni
2) ambosessi
3) Diagnosi di esiti di stroke avvenuto tra 2 mesi e 18 mesi precedenti all'arruolamento
4) Alterazione della funzionalità dell'arto superiore (Motricity Index con punteggio totale dell'arto superiore ≤ 80)
5) Alterazione sensomotoria e propriocettiva dell'arto superiore (Thumb Location Test negativo in 3 prove su 4)
6) Firma del consenso informato alla partecipazione allo studio.

Criteri di esclusione:

1) Instabilità clinica in atto
2) Comorbidità psichiatrica grave che possa interferire con l'adesione al protocollo di trattamento proposto (ad esempio, gravi disturbi della personalità, grave agitazione psicomotoria)
3) Gravi alterazioni della motricità dell'arto superiore : Motricity Index Scale con i seguenti cut-off: presa a pinza < 11, flessione gomito < 14, abduzione spalla < 14
4) Grave deficit di comprensione verbale: Token test (Spinnler 1987) con punteggio < 2
5) grave eminegligenza spaziale : Barrage test (albert 1973) punteggio >3

3.2. Procedure dello studio

I pazienti che rispondono ai criteri di inclusione, in assenza di tutti i criteri di esclusione, verranno arruolati presso l'UO Medicina Riabilitativa e Neuro-riabilitazione (SC) IRCCS Istituto Scienze Neurologiche di Bologna dopo firma del consenso informato. Al fine del reclutamento, il PI (o un suo delegato) illustrerà al paziente il razionale e le modalità operative dello studio utilizzando le informazioni disponibili nella scheda informativa dello studio. Dopo l'arruolamento i pazienti inclusi verranno randomizzati in due gruppi, un gruppo di trattamento (VR) che eseguirà la riabilitazione dell'arto superiore per il recupero propriocettivo e senso-motorio tramite sistema Khymeia utilizzando esercizi in realtà virtuale, e un gruppo di controllo (C) che eseguirà la riabilitazione dell'arto superiore come da normale pratica clinica . Entrambi i gruppi riceveranno lo stesso numero di sedute riabilitative con la medesima durata e cadenza settimanale per il recupero senso-motorio e propriocettivo dell'arto superiore. Qualora i pazienti inclusi avessero necessità di eseguire un protocollo

riabilitativo per il recupero di altri deficit correlati all’evento ictale (es: disturbi del linguaggio, disfagia, emiplegia degli arti inferiori, alterazioni della deambulazione) viene garantito l’esecuzione del percorso riabilitativo come da normale pratica clinica per tali disturbi.

La lista di randomizzazione verrà generata dal PI prima dell’inizio dello studio tramite Microsoft Excel e custodita da uno sperimentatore che non si occuperà della valutazione dei pazienti. I pazienti verranno assegnati ai due gruppi seguendo la sequenza di randomizzazione.

Successivamente i pazienti di entrambi i gruppi saranno sottoposti alle valutazioni pre-test (T0).

3.3. Gruppo trattamento (VR)

Verrà impostato dal team riabilitativo (medico, logopedista, fisioterapista e psicologo) un protocollo riabilitativo individuale per il recupero senso-motorio e propriocettivo dell’arto superiore caratterizzato da esercizi di exergaming con realtà virtuale per una durata complessiva di 50 minuti per 3 giorni alla settimana per 4 settimane consecutive. Il protocollo di riabilitazione con realtà virtuale verrà svolto tramite il sistema VRRS Khymeia , nel quale verranno selezionati esercizi di realtà virtuale semi-immersiva (attraverso l’uso del sistema VRRS HandBox, un avanzato device di skeleton tracking dell’arto superiore) ed esercizi di realtà virtuale immersiva (realizzata con dispositivo Head Mounted Display).

La riabilitazione sarà effettuata presso l’UOC Medicina Riabilitativa e Neuro-riabilitazione IRCCS Istituto Scienze Neurologiche di Bologna.

3.4. Gruppo di controllo (C)

I pazienti del gruppo di controllo svolgeranno il trattamento riabilitativo per il deficit senso-motorio e propriocettivo dell’arto superiore come da normale pratica clinica, con sedute tri-settimanali della durata di 50 minuti per 4 settimane.

Al termine delle 4 settimane di trattamento, i pazienti di entrambi i gruppi saranno sottoposti a valutazioni post- test (T1)

3.5. Valutazioni pre-test

a) Motricity Index Test (Bohannon, 1999)
b) Fugl-Meyer Test (Fugl-Meyer et al., 1975)
c) Box and Block Test (Mathiowetz et al., 1985)
d) Functional Indipendence Measure (FIM) (Beninato et al., 2006)

e) Multidimensional Assessment of Interoceptive Awareness (MAIA) (Machorrinho et al., 2019)
f) Thumb Location Test (Rand, 2018)
g) Rubber Hand Illusion test (Romano et al., 2021)
h) STIMA test (Tessari et al., 2015)

i) Intelligenza generale: Raven Progressive Matrices PM47 (Carlesimo et al., 1996);
j) Funzioni esecutive: Trail Making Test, parte A (attenzione) e B (Giovagnoli et al., 1996);
k) Memoria: 15 words memory test (Ray, 1994), Corsi spatial short-term memory (Spinnler, 1987), digit span for verbal short-term memory digit (Orsini et al., 1987);
l) Abilità visuo-spaziali: VOSP, Visual Object and Space Perception battery; shape detection and shape decision (Warrington & James, 1991)
m) Attenzione: trial making test, matrici attentive, fluenza alternata.

3.6. Valutazioni post-test

a) Motricity Index Test (Bohannon, 1999)
b) Fugl-Meyer Test (Fugl-Meyer et al., 1975)
c) Box and Block Test (Mathiowetz et al., 1985)
d) Functional Indipendence Measure (FIM) (Beninato et al., 2006)
e) Multidimensional Assessment of Interoceptive Awareness (MAIA) (Machorrinho et al., 2019)
f) Thumb Location Test (Rand, 2018) NO
g) Rubber Hand Illusion test (Romano et al., 2021)
h) STIMA test (Tessari et al., 2015) NO

i) Attenzione: trial making test, matrici attentive, fluenza alternata.

j) Client Satisfaction Questionnaire-8

4. Outcome

4.1. Endpoint primario

Al fine di valutare l'effetto che il trattamento proposto genera a livello dell'impairment senso-motorio e propriocettivo dell'arto superiore, viene eseguito un confronto tra le valutazioni basali (T0) e quelle eseguite alla fine delle 4 settimane di trattamento riabilitativo (T1).

4.2. Endpoint secondario:

- Al fine di confrontare l’effetto del trattamento riabilitativo con realtà virtuale per i deficit senso-motori e propriocettivi dell’arto superiore rispetto alla riabilitazione convenzionale, viene eseguito un confronto tra le valutazioni a T0 e T1 tra il gruppo trattato con realtà virtuale (VR) e il gruppo che esegue la riabilitazione come da normale pratica clinica (C)

-Per valutare la soddisfazione del paziente rispetto al trattamento sperimentale proposto, viene somministrato un questionario validato per indagare la soddisfazione del paziente. I dati vengono elaborati calcolando media, mediana, moda e deviazione standard per tutti gli 8 item. In questo modo è possibile identificare quali sono le considerazioni generali del paziente sul trattamento proposto.

5. Aspetti statistici

5.1. Sample size

Si prevede di arruolare in questo studio 20 pazienti, 10 pazienti gruppo sperimentale (VR) e 10 pazienti come controllo (C).

Considerando che la durata dello studio sarà di 24 mesi dei quali gli ultimi 6 saranno dedicati alla elaborazione statistica, analisi dei risultati e disseminazione scientifica, i mesi attivi di reclutamento e inclusione dei pazienti è calcolata in 18 mesi. Considerando che ogni paziente parteciperà allo studio per 6 settimane (1 settimane per la valutazione a T0, 4 settimane per la riabilitazione e 1 settimana per la valutazione a T1), si ritiene che il numero complessivo di 20 pazienti inclusi sia raggiungibile nell’arco di attività dello studio.

Essendo questo uno studio pilota si ritiene che il sample size sia sufficiente al raggiungimento degli obiettivi preposti (considerando un possibile drop out del 20%).

5.2. Analisi statistiche

L’analisi dei dati sarà svolta attraverso il programma Statistical Package for Social Science (SPSS versione 26.0).

I dati preliminari ottenuti dallo studio pilota permetteranno di stimare la variazione nelle misure di esito tra pre test e post test e tra gruppo di trattamento e gruppo di controllo, dando indicazioni per un eventuale trial successivo con reclutamento di campione più ampio.

I dati saranno analizzati in funzione degli obiettivi espressi, ovvero:

- Analisi descrittive del gruppo sperimentale e del gruppo controllo dei dati socio-demografici includendo media e deviazione standard;

- L’effetto del protocollo riabilitativo con realtà virtuale nel gruppo trattato sarà valutato confrontando gli score delle scale di valutazione al pre-test e post-test: la distribuzione dei dati sarà testata tramite test di Shapiro-Wilk. Il test non parametrico di Friedman sarà utilizzato nel caso di distribuzione non normale mentre l’ANOVA sarà utilizzato in caso di distribuzione normale. Il livello di significatività sarà definito per $p \leq 0.05$.

- L’effetto del protocollo riabilitativo in realtà virtuale rispetto al protocollo riabilitativo standard sarà valutato confrontando gli score delle scale a T0 e T1 tra gruppo VR e gruppo C tramite test non parametrico per campioni indipendenti di Mann Whitney.

6. Rilevanza

Qualora le ipotesi sperimentali fossero confermate, l’utilizzo di un sistema di riabilitazione con realtà virtuale potrebbe essere utilizzato nella normale pratica clinica per riabilitare l’integrazione senso-motoria nel paziente con esiti di stroke. Un approccio innovativo con tecniche di realtà virtuale, oltre a poter stimolare l’aderenza al trattamento del paziente, si propone di supportare la riabilitazione propriocettiva e sensomotoria grazie al paradigma dell’illusione del corpo che durante la riabilitazione tradizionale non avviene. Il potenziamento degli aspetti propriocettivi e sensomotori si considerano di fondamentale importanza per la riabilitazione dell’impairment neuromotorio, perciò, agendo tempestivamente con questa tipologia di intervento a seguito dell’evento cerebrovascolare si potrebbe auspicare ad un recupero funzionale più rapido ed efficace.

7. Fattibilità

- La UOC di Medicina Riabilitativa e Neuro-riabilitazione dell’IRCCS Istituto delle Scienze Neurologiche di Bologna coordina la fase riabilitativa sia acuta (con consulenze quotidiane presso la Stroke Unit) sia sub-acuta del PDTA Stroke. Afferiscono quindi quotidianamente pazienti con esiti di Stroke in varie fasi del percorso riabilitativo. Tali pazienti possono avere le caratteristiche per essere inclusi nel progetto.

- La UOC di Medicina Riabilitativa e Neuro-riabilitazione dell’IRCCS Istituto delle Scienze Neurologiche di Bologna dispone del sistema di riabilitazione VRRS KHYMEIA.

- Il dipartimento di Psicologia di Unibo dispone dell’expertise per la valutazione degli aspetti neurocognitivi dei pazienti arruolati e per la somministrazione delle relative scale di valutazione.

- Il dipartimento di Scienze Biomediche e Neuromotorie di Unibo dispone dell’expertise per la valutazione degli aspetti neuromotori e propriocettivi dei pazienti arruolati.

8. Aspetti etici ed amministrativi

8.1. Consenso informato

Ad ogni potenziale partecipante sarà spiegato in modo esaustivo lo svolgimento dello studio e sarà data la possibilità di fare domande e ricevere risposte ad ogni suo dubbio. Il modulo di consenso informato deve essere firmato dal partecipante o da un suo rappresentante legalmente autorizzato prima della sua partecipazione ad una qualunque delle procedure previste dallo studio. La documentazione relativa al paziente deve essere in grado di dimostrare che il consenso è stato espresso prima della partecipazione allo studio. Una copia del modulo di consenso informato deve essere lasciata al paziente. Il modulo di consenso, firmato dal paziente, deve essere conservato al sito.

8.2. Approvazione del comitato etico

Lo studio sarà condotto in accordo con gli standard internazionali ISO 14:155, con la Good Clinical Practice e in conformità ai principi etici della Dichiarazione di Helsinki (64th WMA General Assembly, Fortaleza, Brazil, 2013). Questo protocollo, il modulo di consenso informato e tutte le informazioni rilevanti necessarie correlate allo studio devono saranno sottoposte al Comitato Etico di riferimento per valutazione e approvazione prima dell'inizio dello studio. Il presente protocollo è privo di rischi per la salute dei partecipanti. Eventuali modifiche al protocollo saranno proposte al Comitato Etico Locale come richiesta di emendamento.

8.3. Confidenzialità dei dati e proprietà dei risultati

Un'adeguata documentazione sarà mantenuta per tutto ciò che riguarda i dati clinici del paziente, i fogli di lavoro, gli appunti infermieristici, i moduli di notifica di evento avverso e di interruzione dello studio. I dati personali saranno conservati in ottemperanza alle indicazioni del GDPR. I risultati saranno presentati esclusivamente in forma aggregata e anonima.

9. Pubblicazione dei risultati

Lo sperimentatore principale si impegna a produrre il report finale, pubblicare tutti i dati raccolti come descritto nel protocollo e a garantire che i dati siano riportati responsabilmente e coerentemente.

In particolare, la pubblicazione dei dati. derivanti dal presente studio avverrà indipendentemente dai risultati ottenuti.

La trasmissione o diffusione dei dati, per il tramite di pubblicazioni scientifiche e/o di presentazione a congressi, convegni e seminari, avverrà esclusivamente a seguito dell'elaborazione meramente statistica degli stessi, o comunque in forma assolutamente anonima.

Bibliografia

M. della salute. (2022.). *No Title*. www.salute.gov.it

Beninato, M., Gill-Body, K. M., Salles, S., Stark, P. C., Black-Schaffer, R. M., & Stein, J. (2006). Determination of the minimal clinically important difference in the FIM instrument in patients with stroke. *Archives of Physical Medicine and Rehabilitation*, *87*(1), 32–39.

Bohannon, R. W. (1999). Motricity index scores are valid indicators of paretic upper extremity strength following stroke. *Journal of Physical Therapy Science*, *11*(2), 59–61.

Cha, K., Wang, J., Li, Y., Shen, L., Chen, Z., & Long, J. (2021). A novel upper-limb tracking system in a virtual environment for stroke rehabilitation. *Journal of Neuroengineering and Rehabilitation*, *18*, 1–13. https://doi.org/http://dx.doi.org/10.1186/s12984-021-00957-6

Duncan, P. W., Goldstein, L. B., Horner, R. D., Landsman, P. B., Samsa, G. P., & Matchar, D. B. (1994). Similar motor recovery of upper and lower extremities after stroke. *Stroke*, *25*(6), 1181–1188. https://doi.org/10.1161/01.STR.25.6.1181

Explained, E. S. (2022). (n.d.). *No Title*. https://ec.europa.eu/eurostat/statistics-explained/index.php?title=Cardiovascular_diseases_statistics

Ezendam, D., Bongers, R. M., & Jannink, M. J. A. (2009). Systematic review of the effectiveness of mirror therapy in upper extremity function. *Disability and Rehabilitation*, *31*(26), 2135–2149. https://doi.org/10.3109/09638280902887768

Fugl-Meyer, A. R., Jääskö, L., Leyman, I., Olsson, S., & Steglind, S. (1975). A method for evaluation of physical performance. *Scand J Rehabil Med*, *7*, 13–31.

Henderson, A., Korner-Bitensky, N., & Levin, M. (2007). Virtual reality in stroke rehabilitation: a systematic review of its effectiveness for upper limb motor recovery. *Topics in Stroke Rehabilitation*, *14*(2), 52–61.

Holden, M. K., Dyar, T. A., & Dayan-Cimadoro, L. (2007). Telerehabilitation using a virtual environment improves upper extremity function in patients with stroke. *IEEE Transactions on Neural Systems and Rehabilitation Engineering*, *15*(1), 36–42.

Hsu, H.-Y., Kuo, L.-C., Lin, Y.-C., Su, F.-C., Yang, T.-H., & Lin, C.-W. (2022). Effects of a Virtual Reality–Based Mirror Therapy Program on Improving Sensorimotor Function of Hands in Chronic Stroke Patients: A Randomized Controlled Trial. *Neurorehabilitation and Neural Repair*, 15459683221081430.

Kilteni, K., Groten, R., & Slater, M. (2012). The Sense of Embodiment in Virtual Reality. *PRESENCE-TELEOPERATORS AND VIRTUAL ENVIRONMENTS*, *21*(4), 373–387. https://doi.org/10.1162/PRES_a_00124 WE - Science Citation Index Expanded (SCI-EXPANDED) WE - Social Science Citation Index (SSCI)

Kim, W.-S., Cho, S., Ku, J., Kim, Y., Lee, K., Hwang, H.-J., & Paik, N.-J. (2020). Clinical

application of virtual reality for upper limb motor rehabilitation in stroke: review of technologies and clinical evidence. *Journal of Clinical Medicine*, *9*(10), 3369.

Langhorne, P., Coupar, F., & Pollock, A. (2009). Motor recovery after stroke: a systematic review. *The Lancet Neurology*, *8*(8), 741–754.

Machorrinho, J., Veiga, G., Fernandes, J., Mehling, W., & Marmeleira, J. (2019). Multidimensional assessment of interoceptive awareness: Psychometric properties of the Portuguese version. *Perceptual and Motor Skills*, *126*(1), 87–105.

Mathiowetz, V., Volland, G., Kashman, N., & Weber, K. (1985). Adult norms for the Box and Block Test of manual dexterity. *The American Journal of Occupational Therapy*, *39*(6), 386–391.

Pollock, A., Farmer, S. E., Brady, M. C., Langhorne, P., Mead, G. E., Mehrholz, J., & van Wijck, F. (2014). Interventions for improving upper limb function after stroke. *Cochrane Database of Systematic Reviews*, *11*.

Rand, D. (2018). Proprioception deficits in chronic stroke—Upper extremity function and daily living. *PloS One*, *13*(3), e0195043.

Romano, D., Maravita, A., & Perugini, M. (2021). Psychometric properties of the embodiment scale for the rubber hand illusion and its relation with individual differences. *SCIENTIFIC REPORTS*, *11*(1). https://doi.org/10.1038/s41598-021-84595-x WE - Science Citation Index Expanded (SCI-EXPANDED) WE - Social Science Citation Index (SSCI)

Steenbergen, B., & Van Der Kamp, J. (2004). Control of prehension in hemiparetic cerebral palsy: similarities and differences between the ipsi-and contra-lesional sides of the body. *Developmental Medicine and Child Neurology*, *46*(5), 325–332.

Tambone, R., Giachero, A., Calati, M., Molo, M. T., Burin, D., Pyasik, M., Cabria, F., & Pia, L. (2021). Using Body Ownership to Modulate the Motor System in Stroke Patients. *Psychological Science*, *32*(5), 655–667. https://doi.org/10.1177/0956797620975774

Tessari, A., Toraldo, A., Lunardelli, A., Zadini, A., & Rumiati, R. I. (2015). STIMA: a short screening test for ideo-motor apraxia, selective for action meaning and bodily district. *Neurological Sciences*, *36*(6), 977–984.

Tosi, G., Romano, D., & Maravita, A. (2018). Mirror box training in hemiplegic stroke patients affects body representation. *Frontiers in Human Neuroscience*, *11*, 617.

Turolla, A., Dam, M., Ventura, L., Tonin, P., Agostini, M., Zucconi, C., Kiper, P., Cagnin, A., & Piron, L. (2013). Virtual reality for the rehabilitation of the upper limb motor function after stroke: a prospective controlled trial. *Journal of Neuroengineering and Rehabilitation*, *10*(1), 1–9.

Wallwork, S. B., Bellan, V., Catley, M. J., & Moseley, G. L. (2016). Neural representations and the cortical body matrix: implications for sports medicine and future directions. *British Journal of Sports Medicine*, *50*(16), 990–996.

Weber, L. M., Nilsen, D. M., Gillen, G., Yoon, J., & Stein, J. (2019). Immersive virtual reality mirror therapy for upper limb recovery following stroke: A pilot study. *American Journal of Physical Medicine & Rehabilitation*, *98*(9), 783.
